# Prevalence and determinants of serum antibodies to SARS-CoV-2 in the general population of the Gardena valley

**DOI:** 10.1017/S0950268821001886

**Published:** 2021-08-03

**Authors:** Roberto Melotti, Federica Scaggiante, Michela Falciani, Christian X. Weichenberger, Luisa Foco, Stefano Lombardo, Alessandro De Grandi, Dorothee von Laer, Angelika Mahlknecht, Peter P. Pramstaller, Elisabetta Pagani, Horand Meier, Timon Gaertner, Christina Troi, Deborah Mascalzoni, Cristian Pattaro, Michael Mian

**Affiliations:** 1 Institute for Biomedicine (affiliated to the University of Lübeck), Eurac Research, Bolzano, Italy; 2 Laboratorio di Patologia Clinica di Bressanone, Hospital of Bressanone (SABES-ASDAA), Bressanone-Brixen, Italy; 3 Servizio farmaceutico, Hospital of Bolzano (SABES-ASDAA), Bolzano-Bozen, Italy; 4 Istituto provinciale di Statistica (ASTAT), Amministrazione Provincia Bolzano, Bolzano, Italy; 5 Institute of Virology, Department of Hygiene, Microbiology and Public Health at Innsbruck Medical University, Innsbruck, Austria; 6 Istituto di Medicina Generale, Scuola Provinciale Superiore di Sanità (Claudiana), Bolzano, Italy; 7 Institute of General Practice, Family Medicine and Preventive Medicine, Paracelsus Medical University, Salzburg, Austria; 8 Laboratorio Aziendale di Microbiologia e Virologia, Hospital of Bolzano (SABES-ASDAA), Bolzano-Bozen, Italy; 9 Unità operativa governo clinico, Amministrazione Provincia Bolzano, Bolzano, Italy; 10 Centre for Research Ethics and Bioethics (CRB), Department for Public Health and Caring Sciences, Uppsala University, Uppsala, Sweden; 11 Service for Innovation, Research and Teaching, Hospital of Bolzano (SABES-ASDAA), Bolzano-Bozen, Italy; 12 Scuola Provinciale Superiore di Sanità (Claudiana), Bolzano, Italy

**Keywords:** Coronavirus, COVID-19, neutralising antibodies, prevalence, SARS-CoV-2

## Abstract

Estimating the spread of SARS-CoV-2 infection in communities is critical. We surveyed 2244 stratified random sample community members of the Gardena valley, a winter touristic area, amidst the first expansion phase of the COVID-19 pandemic in Europe. We measured agreement between Diasorin and Abbott serum bioassay outputs and the Abbott optimal discriminant threshold of serum neutralisation titres with recursive receiver operating characteristic curve. We analytically adjusted serum antibody tests for unbiased seroprevalence estimate and analysed the determinants of infection with non-response weighted multiple logistic regression. SARS-CoV-2 seroprevalence was 26.9% (95% CI 25.2–28.6) by June 2020. The bioassays had a modest agreement with each other. At a lower threshold than the manufacturer's recommended level, the Abbott assay reflected greater discrimination of serum neutralisation capacity. Seropositivity was associated with place and economic activity, not with sex or age. Symptoms like fever and weakness were age-dependent. SARS-CoV-2 mitigation strategies should account for context in high prevalence areas.

## Introduction

During the initial phase of the SARS-CoV-2 pandemic, the Gardena valley, a well-known winter tourism destination located in South Tyrol (Italy), became one of the European regions most afflicted by the coronavirus disease 2019 (COVID-19). While in the middle of the virus circulation vortex since February 2020, there were a multitude of holidaymakers and visitors in the valley mainly from Northern Italy and Central Europe. Back home, tourists likely contributed to further transmission of the virus just before containment actions were endorsed by regions worldwide [1].

As expected in such an emergent phase of the pandemic, hospital-based case reports dominated the accumulation of scientific evidence on COVID-19 [2]. Consequently, public awareness, ongoing knowledge of the determinants of disease and disease severity, and current prevention strategies have been profoundly influenced by clinical observations, while evidence from community studies has had limited space in context [3]. Specific knowledge of the exogenous determinants of SARS-CoV-2 infection and its related symptoms or about biological susceptibility in the general population is still incomplete, probably due to the slower pace and relative paucity of community-based studies [4]. Geographically confined regions with a relatively high incidence of infection may help characterise the spread of COVID-19, providing useful indications to policy-makers for current and future preventive efforts.

At the end of May 2020, we surveyed 2244 inhabitants of the Gardena valley representative of the local population, measured antibody test response to SARS-CoV-2 and related that response to symptoms, prior conditions and serum neutralisation capacity. The high seroprevalence qualified the in-depth analysis of determinants and COVID-19-related symptoms in a general population setting, augmenting the general understanding of the disease dynamic.

## Methods

### Study design

Invited to the study were 2958 of the 9424 inhabitants of Ortisei, Santa Cristina and Selva, the main municipalities of the Gardena valley, following a one-stage random sampling design stratified by municipality, sex and age group (<6, 6–17, 18–34, 35–49, 50–64, 65+ years). Sample size was defined based on an expected 3% minimal seroprevalence with 0.25% relative standard error (s.e.) and accounting for finite population correction. Participants were selected with known extraction probability from the municipality registries, excluding nursing homes, using the ‘surveyselect’ program in SAS v9.2.

Participants were invited via letter including the planned participation date; a link to the online questionnaire (with telephone support) covering demographic, clinical and socio-behavioural aspects (Supplementary Material page 2); a personalised password for use as pseudo-anonymisation code. Testing procedures included a nasopharyngeal swab test and a serological antibody test (limited to 6+ years old participants). The study took place between 26 May and 8 June 2020. The Ethics Committee of the Healthcare System of the Autonomous Province of Bolzano-Bozen authorised the study. Each participant gave written informed consent.

### Biological sample collection and analysis

Swab samples were analysed at the ÖNORM-accredited (EN ISO 15189:2013) diagnostic laboratory of the Institute of Virology of the Innsbruck Medical University (IVIMU, Austria) as described in the Supplementary Material page 7. As no molecular test was available at the time of primary infection and no swab sample tested positive at enrolment to the study, this analysis was not considered further.

Antibody response was tested using the Abbott SARS-CoV-2 IgG assay (Sligo, Ireland), designed to detect immunoglobulin class G (IgG) antibodies to the nucleocapsid (N) protein of SARS-CoV-2. Fresh serum samples were collected in blood tubes with separating gel. Within 6 h from collection, assessment of IgG antibodies to SARS-CoV-2 was performed using the Abbott Architect i2000SR system, which implements a two-step chemiluminescent microparticle immunoassay, at the Laboratory of Clinical Pathology of the Bressanone-Brixen Hospital, Italy. Seropositivity was defined as a signal-to-calibrator (S/C) Abbott Architect Index (AAI) value ≥1.4. At this threshold, the manufacturer reported 96.9% (89.5–99.5%) sensitivity at 14 days after symptoms onset, 100.0% (95.1–100.0%) sensitivity at 17 days and 99.9% specificity [5]. Biological samples of study participants were stored at the Eurac Research Biobank (ERB) at the Bolzano-Bozen Hospital, Italy, as described in the Supplementary Material page 7.

Two hundred and ninety-nine serum samples were selected for plaque reduction neutralisation test (PRNT) [6], ensuring the coverage of the whole SARS-CoV-2 IgG assay AAI distribution while maximising sample heterogeneity in terms of sex, age and symptoms manifestation as well as previous diagnosis and hospitalisation (additional details in Supplementary Material page 7 and Supplementary Fig. S1A and S1B). Selected frozen serum samples were dry ice shipped from the ERB to the IVIMU diagnostic laboratory. After 30 min heat inactivation at 56 °C, samples were table top centrifuged for 5 min at 8000 rpm. They were fourfold serially diluted in complete medium containing 2% foetal calf serum (FCS) starting with a 1:4 dilution in duplicate samples. Serum dilutions were mixed with an equal volume of a replication-competent SARS-CoV-2 resulting in ~300 infected cells in non-neutralised wells. Serum-virus mixes were incubated for 1 h at 37 °C and subsequently transferred to 96-wells containing 90% confluent Vero cells expressing TMPRSS2 seeded one day before. Cells were infected with the virus for 1 h at 37 °C and subsequently washed once with complete medium with 2% FCS. After adding fresh complete medium containing 2% FCS, cells were further cultured for 13 h. Cells were fixed for 5 min with 96% ethanol and subsequently stained using the serum from a SARS-CoV-2 recovered patient and a horseradish peroxidase-conjugated anti-human secondary antibody (Dianova). Plates were developed using 3-amino-9-ethylcarbazole substrate. Infected cells were counted via microscope and 50% neutralisation titres were calculated as the highest dilution where the mean infection of duplicate samples was reduced by >50% of the mean of control wells lacking the virus.

The presence of spike (S) protein antibodies in the 299 samples was assessed with the Diasorin LIAISON® SARS-CoV-2 S1/S2 IgG chemiluminescent assay (Saluggia, Italy), designed to detect the number of arbitrary units (AUs) of specific IgG class antibodies directed against the S1 and S2 viral proteins, at the Microbiology and Virology Laboratory of the Healthcare System of the Autonomous Province of Bolzano-Bozen (Bolzano-Bozen, Italy). Results were classified as negative (values <12 AU/ml), inconclusive (values ≥12 and <15 AU/ml) or positive (values ≥15 AU/ml) according to the manufacturer's recommendations. This assay had declared 97.4% sensitivity >15 days after symptoms onset, and 98.9% specificity [7].

### Statistical analyses

We assessed pairwise agreement between quantitative variables with the Lin's concordance correlation coefficient (CCC) [8] and Bland–Altman plot [9], and agreement between categorical variables using the Cohen's *κ* statistic [10], using the ‘epiR’ v1.0-15, ‘BlandAltmanLeh’ v0.3.1 and ‘psych’ v1.9.11 packages in the R software v3.3.6.

To investigate the discrimination accuracy of the 1.4 AAI threshold on thawed serum against a PRNT value ≥4, which was considered as a gold-standard for prior exposure to SARS-CoV-2, we conducted receiver operating characteristic (ROC) curve analysis identifying the optimal cut-off as the Youden's index (*J*) [11]. Overfitting was prevented by performing both 10-fold cross-validation and repeated random sub-sampling validation. In the latter approach, we randomly split the sample set into 80% and 20% training and test sets, respectively, corresponding to 239 and 60 observations, repeatedly 5000 times. The two validation procedures resulted in sets of 10 and 5000 optimal cut-offs, respectively. We reported the median optimal cut-off of each respective validation procedure.

Descriptive tables and multiple logistic regression models display observed counts for each relevant category, while accounting for the study design: stratification by sex, municipality and age group, post-stratification (citizenship by municipality) and finite population correction, for efficient proportion estimations. To correct for possible selection biases, we also adjusted the sampling weights based on proportional allocation within strata by non-response (balanced to the population strata distribution), which were then calibrated dynamically by post-stratification in each analysis. The prevalence of serum antibodies to SARS-CoV-2 (seroprevalence) in the overall sample was also estimated using the Rogan and Gladen's formula to account for the serological test inaccuracy [12]. Statistical analyses were run with the ‘svyset’ suite of commands in Stata software v16.1 (StataCorp LLC) and were restricted to 6+ years old individuals and non-pregnant women using the ‘subpop’ option.

## Results

Of 2958 invited individuals, 2244 (75.9%) joined the study. Among these, swab and serum antibody test results were available for 2083 (82.8%) and 2129 (94.9%) participants, respectively. Excluding pregnant women and <6-year-old children left 2106 (93.9%) participants for seroprevalence analysis, of whom 1813 (80.8%) filled in the questionnaire-based interview. Participants were balanced across sexes and age groups (Table 1). In terms of sex, age and municipality distribution, the sample was representative of the reference population. Of the 2106 participants undergoing the serum antibody test, 551 tested positive, corresponding to a corrected seroprevalence of 26.9% (95% confidence interval (95% CI) 25.2–28.6%), with no evidence of different seroprevalence between questionnaire completers and non-completers (Supplementary Table S1).

**Table 1.** Socio-demographic characteristics of the 2106 participants with available antibody test results who were considered for the seroprevalence analysis (age 6+; non-pregnant women)

Characteristic	Category/statistics	*N*	% or mean (95% CI)
Sex	Male	1027	48.9 (48.6–49.2)
	Female	1079	51.1 (50.8–51.4)
Municipality	Ortisei	1074	51.2 (51.1–51.3)
	Santa Cristina	455	21.3 (21.2–21.4)
	Selva	577	27.5 (27.5–27.6)
Citizenship	Italian	2004	92.7 (92.6–92.7)
	Other	102	7.3 (7.3–7.4)
Education	Primary school or no title	242	13.2 (12.0–14.5)
	Lower secondary school	397	21.4 (19.9–23.1)
	Vocational school	516	28.3 (26.5–30.1)
	Upper secondary school	463	25.8 (24.1–27.5)
	Higher degree	195	11.3 (10.0–12.6)
Language	German	312	17.6 (16.1–19.3)
	Italian	161	9.2 (8.0–10.5)
	Ladin	1274	67.5 (65.7–69.2)
	Other	66	5.7 (4.9–6.7)
Economic activity^a^	Accommodation and catering	399	22.8 (21.2–24.5)
	Healthcare and social services	59	3.3 (2.6–4.0)
	Other activity	586	32.4 (30.7–34.1)
	Student or not active	769	41.6 (40.0–43.1)
Age-class	06–17	284	13.1 (12.9–13.3)
	18–34	440	21.9 (21.6–22.1)
	35–49	403	20.9 (20.5–21.2)
	50–64	519	23.0 (22.9–23.2)
	65–99	460	21.2 (21.0–21.4)
Age	Mean	2106	45.1 (44.9–45.4)
BMI^a^	Mean	1797	23.2 (23.0–23.3)
Serological test	Negative	1555	73.7 (72.0–75.4)
	Positive	551	26.3 (24.6–28.0)

Figures are corrected for the survey design settings (see Methods).

a Frequency distribution of economic activity and mean of body mass index (BMI) obtained on 1813 and 1797 participants, respectively, who also filled in the questionnaire.

### Comparison of testing methods and plaque reduction neutralisation test

**Fig. 1.**
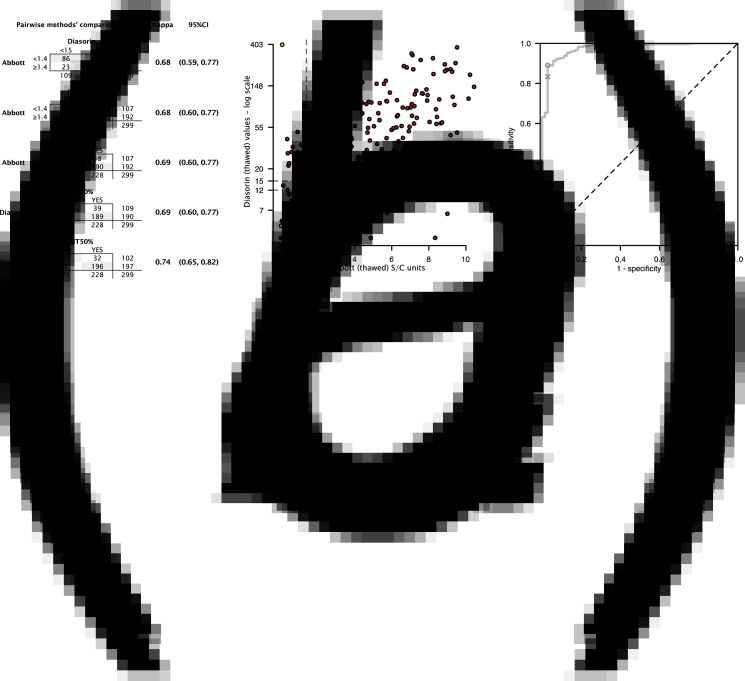
Antibody test performance evaluations. (a) Pairwise comparisons and *κ* statistics with their 95% CIs across all antibody assays and PRNT at 50%. (b) Scatterplot of the Abbott assay results (*x*-axis) *vs.* the Diasorin assay results (*y*-axis, in logarithmic scale), in the context of PRNT results (yellow dots: negative; brown dots: positive). Dashed lines indicate the clinically relevant thresholds for positivity. (c) ROC curve used to define the optimal cut-off for S/C (AAI) values as a classifier. Plotted here is the true-positive rate (sensitivity) *vs.* the false-positive rate (1 – specificity) for increasing values of AAI for all 299 individuals subject to PRNT. The diagonal corresponds to the ROC curve of a random classifier. Discriminative classifiers produce curves drawn towards the upper left corner, where sensitivity = specificity = 1. A perfectly discriminating classifier generates a ROC curve that starts at the lower left corner and advances as a vertical line to the upper left corner and from there horizontally to the upper right corner. The cross corresponds to the classifier performance using AAI = 1.4. In the sample under investigation, optimal classifier performance is achieved for AAI = 1.16 (circle).

The 299 participants' samples chosen for PRNT analysis had similar characteristics to the whole sample (Supplementary Table S2). Their S/C (AAI) values were uniformly distributed across the whole range observed (Supplementary Fig. S1C), resulting in 194 seropositive samples. To exclude sample logistic, handling and thawing procedure effects, we first compared Abbott antibody test results in fresh against post-conservation thawed serum, observing almost perfect agreement (CCC = 0.9982; 95% CI 0.9978–0.9986; Supplementary Fig. S2A) despite minimal discordance attributable to storage time (Supplementary Fig. S2B) and plate (Supplementary Fig. S2C) effects. Post-thawing sample levels were on average −0.08 (95% CI −0.09 to −0.06) AAI units lower than those observed in fresh blood (Supplementary Fig. S2B), with two positive samples reclassified as negative (*κ* = 0.99; 95% CI 0.97–1.00). We observed 192 (64.2%) positive samples by the Abbott assay at the 1.4 canonical threshold, and 190 (63.5%) and 197 (65.9%) positive samples by the Diasorin test at the 15 and 12 thresholds, respectively (Supplementary Table S2, Fig. S1A). In either case, the concordance between Abbott and Diasorin assays was limited (Fig. 1a and 1b).

Of the 299 samples, 228 (76.3%) showed 50% neutralisation capacity. This was in limited agreement with both Abbott (*κ* = 0.69; 95% CI 0.60–0.77) and Diasorin assay when evaluated at the canonical threshold (*κ* = 0.69; 95% CI 0.60–0.77; Fig. 1a). Higher agreement was observed between PRNT and Diasorin test when the latter was evaluated at a threshold of 12 (*κ* = 0.74; 95% CI 0.65–0.82). In any case, of the 83 samples testing negative with both Abbott (<1.4) and Diasorin (<12) assays, 15 (18.1%) showed neutralisation capacity. In contrast, neutralisation capacity was always confirmed when both the Abbott and Diasorin tests were positive, and almost always when at least one of them was positive (Fig. 1b).

The optimal Abbott antibody test threshold identified in the ROC curve analysis was 1.16 (Fig. 1c), consistently in both the 10-fold cross-validation and 5000 repeated random sub-sampling validation, both returning 1.16 median AAI. At this threshold, the classifier performed with 89.0% sensitivity and 97.2% specificity. At the recommended 1.4 threshold, sensitivity was lower (83.3%) but specificity was the same. Sex and age stratification improved the assay performance with relatively lower thresholds but at the cost of reliability (Supplementary Fig. S3, Table S3).

### Identification of determinants and predictors of seropositivity

Among all socio-demographic and lifestyle characteristics considered (Table 1), being a current smoker (OR 0.35; 95% CI 0.25–0.49), working in the accommodation and catering services (OR 1.37; 95% CI 1.05–1.77), living in Selva (OR 1.37; 95% CI 1.10–1.70) or Santa Cristina (OR 1.31; 95% CI 1.03–1.68) and having higher BMI (OR 1.14; 95% CI 1.02–1.28; for every standard deviation increase) were each individually and independently associated with positivity to the Abbott antibody test (Table 2). Females did appear at lower risk than males; however, evidence was weak in the mutually adjusted analysis.

**Table 2.** Prevalence of serum antibodies to SARS-CoV-2 and participants' socio-demographic characteristics (*N* = 1813)

Characteristic	Category/statistics	*N*	Seroprevalence % (95% CI)	*P*-value^a^	adjusted *P*-value^b^	OR (95% CI)^c^	OR adjusted *P*-value^c^
Sex	Male	884	28.3 (25.8–31.0)	0.013	0.070	Ref.	
	Female	929	23.8 (21.5–26.3)			0.83 (0.68–1.02)	0.070
Smoking habit	Non-smoker	1278	27.6 (25.5–29.8)	<0.001	<0.001	Ref.	
	Past smoker	273	31.2 (26.6–36.3)			1.07 (0.82–1.39)	0.609
	Current smoker	262	13.1 (9.8–17.1)			0.35 (0.25–0.49)	<0.001
Main activity	Accomodation and catering	399	31.6 (27.7–35.8)	0.002	<0.001	1.37 (1.05–1.77)	0.019
	Healthcare and social services	59	26.5 (17.9–37.4)			1.26 (0.72–2.20)	0.425
	Other activity	586	26.5 (23.5–29.8)			Ref.	
	Student or not active	769	22.5 (20.0–25.2)			0.82 (0.65–1.03)	0.089
Municipality	Ortisei	908	22.6 (20.3–25.1)	<0.001	0.010	Ref.	
	Santa Cristina	389	28.3 (24.5–32.5)			1.31 (1.03–1.68)	0.031
	Selva	516	30.2 (26.9–33.9)			1.37 (1.10–1.70)	0.006
Nationality	Italian	1744	26.1 (24.3–27.9)	0.829	0.692	Ref.	
	Other	69	25.1 (17.1–35.3)			0.90 (0.53–1.53)	0.692
Education	Primary school or no title	242	23.9 (19.5–28.9)	0.889	0.529	Ref.	
	Lower secondary school	397	27.0 (23.4–31.1)			1.19 (0.84–1.68)	0.320
	Vocational school	516	26.6 (23.3–30.1)			1.02 (0.72–1.44)	0.932
	Upper secondary school	463	25.7 (22.4–29.4)			0.97 (0.68–1.39)	0.877
	Higher degree	195	25.7 (20.6–31.6)			0.89 (0.57–1.37)	0.590
Language	German	312	23.6 (19.7–28.1)	0.379	0.454	Ref.	
	Italian	161	24.7 (19.3–31.2)			0.88 (0.59–1.32)	0.544
	Ladin	1274	27.1 (25.0–29.3)			1.09 (0.83–1.42)	0.550
	Other	66	21.7 (14.1–31.9)			0.78 (0.44–1.39)	0.403
Age	Median	1813	26.1 (24.2–27.9)	0.168	0.827	Ref.	
	Median + 5 years	1813	26.3 (24.5–28.2)			1.00 (0.98–1.03)	0.827
BMI^d^	Mean	1797	25.7 (23.9–27.5)	<0.001	0.023	Ref.	
	Mean + 1 s.d.	1797	28.9 (26.3–31.5)			1.14 (1.02–1.28)	0.023

Seroprevalence figures at specific values of age and BMI were obtained by predictive margins with linearised standard errors from a logistic model. BMI, body mass index; s.d., standard deviation.

a Survey design adjusted *F* statistics. For continuous variables (age, BMI), the *P*-value was obtained by the adjusted Wald test with linearised standard errors.

b Survey design adjusted *F* statistics, multiply adjusted for sex, age, BMI, smoking habit, main activity and municipality. For continuous variables (age, BMI), the *P*-value was obtained by the adjusted Wald test with linearised standard errors.

c Odd ratios (ORs), 95% confidence intervals (95% CIs) and related adjusted *P*-values were obtained by linearised standard errors and logit transformation.

d There were 16 missing values.

Focusing on symptoms as possible seroprevalence predictors, we found strong evidence for any single symptom to predict antibody positivity, both in unadjusted and mutually adjusted analyses, which considered each symptom at one time (Table 3). Seropositivity was 38.8% (95% CI 36.2–41.5) in those reporting any number of symptoms, 45.6% (95% CI 42.3–48.9) in those reporting multiple symptoms, 10.0% (95% CI 8.3–12.0) in those reporting no symptoms (*P* < 0.001, Table 3) and 14.2% (95% CI 12.4–16.1) in those with at most one symptom, respectively (Table 3, Table 4).

**Table 3.** Prevalence of serum antibodies to SARS-CoV-2 by participants' reported symptoms (*N* = 1813)

Symptom	Category	*N*	Seroprevalence % (95% CI)	*P*-value^a^	Adjusted *P*-value^b^	OR (95% CI)^c^	OR adjusted *P*-value^c^
Fever>37.5 °C	No	1581	21.2 (19.5–23.1)	<0.001	<0.001	Ref.	
	Yes	232	58.4 (52.7–63.8)			5.52 (4.26–7.15)	<0.001
Cough	No	1426	22.0 (20.2–24.0)	<0.001	<0.001	Ref.	
	Yes	387	40.5 (36.3–44.9)			2.32 (1.87–2.87)	<0.001
Sorethroat or cold symptoms	No	1398	23.4 (21.5–25.5)	<0.001	<0.001	Ref.	
	Yes	415	34.4 (30.5–38.6)			1.71 (1.37–2.12)	<0.001
Headache	No	1406	21.9 (20.0–23.8)	<0.001	<0.001	Ref.	
	Yes	407	40.1 (35.9–44.3)			2.37 (1.91–2.95)	<0.001
Pain in the limbs	No	1452	18.8 (17.1–20.7)	<0.001	<0.001	Ref.	
	Yes	361	55.1 (50.5–59.6)			5.45 (4.35–6.82)	<0.001
Loss of taste/smell	No	1588	18.6 (17.0–20.4)	<0.001	<0.001	Ref.	
	Yes	225	77.3 (72.1–81.7)			15.05 (11.18–20.26)	<0.001
Difficulty in breathing	No	1731	24.2 (22.4–26.0)	<0.001	<0.001	Ref.	
	Yes	82	64.0 (54.4–72.6)			5.47 (3.68–8.13)	<0.001
Chest pain	No	1697	24.6 (22.8–26.4)	<0.001	<0.001	Ref.	
	Yes	116	46.5 (38.6–54.5)			2.41 (1.71–3.40)	<0.001
Increased pulse rate	No	1779	25.5 (23.8–27.3)	<0.001	<0.001	Ref.	
	Yes	34	51.5 (36.9–65.8)			2.92 (1.63–5.21)	<0.001
Gastrointestinal problems	No	1597	23.6 (21.8–25.4)	<0.001	<0.001	Ref.	
	Yes	216	43.9 (38.2–49.8)			2.73 (2.09–3.56)	<0.001
Conjunctivities	No	1724	25.2 (23.4–27.0)	<0.001	<0.001	Ref.	
	Yes	89	42.2 (33.4–51.5)			2.12 (1.44–3.13)	<0.001
Weakness	No	1539	20.9 (19.1–22.7)	<0.001	<0.001	Ref.	
	Yes	274	54.7 (49.5–59.8)			4.66 (3.66–5.94)	<0.001
Any symptoms	No	811	10.0 (8.3–12.0)	<0.001	<0.001	Ref.	
	Yes	1002	38.8 (36.2–41.5)			5.88 (4.61–7.50)	<0.001
Symptom count	None	811	10.0 (8.3–12.0)	<0.001	<0.001	Ref.	
	Any	322	24.6 (20.6–29.0)			2.95 (2.15–4.04)	<0.001
	Multiple (2+)	680	45.6 (42.3–48.9)			7.87 (6.10–10.16)	<0.001

a Survey design adjusted *F* statistics.

b Survey design adjusted *F* statistics, multiply adjusted for sex, age, BMI, smoking habit, main activity and municipality.

c Odd ratios (ORs), 95% confidence intervals (95% CIs) and related adjusted *P*-values are obtained by linearised standard errors and logit transformation.

**Table 4.** Prevalence of serum antibodies to SARS-CoV-2 by participants' reported period of symptoms (*N* = 1813)

	*N*	Seroprevalence % (95% CI)	*P*-value^a^	Adjusted *P*-value^b^	OR (95% CI)^c^	OR adjusted *P*-value^c^
Period of 1+ symptoms onset						
No symptoms	811	10.0 (8.3–12.0)	<0.001	<0.001	Ref.	
Feb: 1st	150	14.2 (10.0–19.7)			1.58 (0.99–2.51)	0.055
Feb: 2nd	197	30.5 (25.1–36.6)			4.01 (2.83–5.68)	<0.001
March: 1st	380	56.7 (52.3–61.0)			12.18 (9.18–16.14)	<0.001
March: 2nd	198	44.2 (38.2–50.3)			7.03 (5.10–9.69)	<0.001
April onwards	77	7.6 (3.8–14.5)			0.77 (0.36–1.64)	0.496
Period of 2+ symptoms onset						
At most 1 symptom	1133	14.2 (12.4–16.1)	<0.001	<0.001	Ref.	
Feb: 1st	103	14.5 (9.6–21.2)			1.10 (0.66–1.83)	0.711
Feb: 2nd	129	35.8 (28.9–43.5)			3.55 (2.46–5.11)	<0.001
March: 1st	278	63.2 (58.1–68.0)			10.81 (8.23–14.20)	<0.001
March: 2nd	137	50.3 (42.9–57.6)			5.93 (4.29–8.20)	<0.001
April onwards	33	14.9 (7.1–28.7)			1.02 (0.44–2.40)	0.955

a Survey design adjusted *F* statistics.

b Survey design adjusted *F* statistics, multiply adjusted for sex, age, BMI, smoking habit, main activity and municipality.

c Odd ratios (ORs), 95% confidence intervals (95% CIs) and related adjusted *P*-values are obtained by linearised standard errors and logit transformation.

**Fig. 2.**
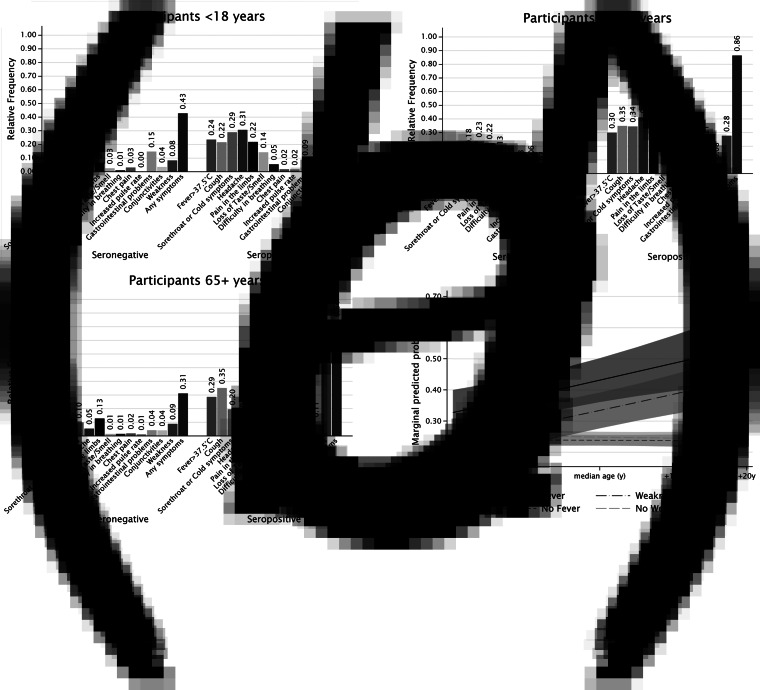
Association between reported symptoms and seroprevalence. (a) to (c) Symptoms frequency distribution in seronegative and seropositive participants, by age group; (a) <18 years old; (b) 18–64 years old; (c) 65+ years old. (d) Marginal predicted probabilities of anti-SARS-CoV-2 IgG antibodies by linear effect of age as moderated by specific symptoms. Linear predictions and 95% confidence bands are displayed on the graph for participants with fever (plain line and dark grey bands), with no fever (dash line and mild grey bands), with weakness (long-dash dot line and plain grey bands) and with no weakness (long-dash line and light grey bands). For example, a participant of median age would have roughly the same marginal probability of infection of either older or younger participants, if they had no symptoms of fever and weakness, integrating across all possible predictors (e.g. *P*_r_ = 0.24 if no fever present and median age, 95% CI 0.22–0.26). However, the estimated marginal probability of infection was 0.33 (95% CI 0.26–0.40) for participants 20 years younger than the median age and 0.54 (95% CI 0.42–0.65) for participants 20 years older than the median age if they had fever. Corresponding probabilities for participants with weakness were 0.24 (95% CI 0.17–0.31) and 0.44 (95% CI 0.36–0.52), for participants 20 years younger and 20 years older than the median age, respectively.

The most predictive symptoms were the loss of taste or smell (OR 15.05; 95% CI 11.18–20.26), fever (OR 5.52; 95% CI 4.26–7.15), difficulty in breathing (OR 5.47; 95% CI 3.68–8.13), pain in the limbs (OR 5.45; 95% CI 4.35–6.82) and weakness (OR 4.66; 95% CI 3.66–5.94). Symptom occurrences were visually different across age groups (Fig. 2a–2c). In multiple logistic regression, we fitted interaction terms of fever and weakness with age (Table 5). The probability of seropositivity was higher in older participants who also reported either fever or weakness (Fig. 2d). However, age was a mild predictor of infection in the absence of fever and weakness and independent of any other symptoms (OR 0.96; 95% CI 0.93–1.00, *P* = 0.046; Table 5).

**Table 5.** Results of the logistic regression model for the association between the prevalence of serum antibodies to SARS-CoV-2 and symptoms while accounting for individual-level relevant characteristics (*n* = 1804)

Predictor	Category/units	OR	95% CI	*P*-value
Sex	Male	Ref.		
Sex	Female	0.90	(0.70–1.15)	0.398
BMI	kg/m^2^	1.04	(1.01–1.07)	0.021
Age	5-yrs units	0.96	(0.93–1.00)	0.046
Symptom	No symptoms	Ref.		
	Fever	3.22	(2.20–4.71)	<0.001
	Fever × age	1.17	(1.07–1.28)	0.001
	Weakness	1.78	(1.23–2.58)	0.002
	Weakness × age	1.19	(1.09–1.29)	<0.001
	Pain in the limbs	2.76	(2.04–3.74)	<0.001
	Loss of taste or smell	10.47	(7.30–15.01)	<0.001
	Difficulty in breathing	1.94	(1.07–3.55)	0.030
	Chest pain	0.53	(0.31–0.90)	0.018
Smoking habit	Never smoker	Ref.		
	Past smoker	0.96	(0.68–1.36)	0.826
	Current smoker	0.28	(0.18–0.44)	<0.001
Main activity	Other activity	Ref.		
	Accommodation and catering	1.40	(1.02–1.94)	0.041
	Healthcare and social services	1.01	(0.55–1.84)	0.987
	Student or not active	1.04	(0.78–1.37)	0.812
Municipality	Ortisei	Ref.		
	Santa Cristina	1.34	(0.99–1.81)	0.061
	Selva	1.38	(1.05–1.82)	0.020
Dietary supplements	No	Ref.		
	Yes	0.51	(0.34–0.75)	0.001
Any drug regularly	No	Ref.		
	Yes	0.64	(0.46–0.89)	0.008

Survey design adjusted odds ratios (ORs) (see Methods). 95% confidence intervals (95% CIs) and related adjusted *P*-values were obtained by linearised standard errors and logit transformation. BMI, body mass index.

**Fig. 3.**
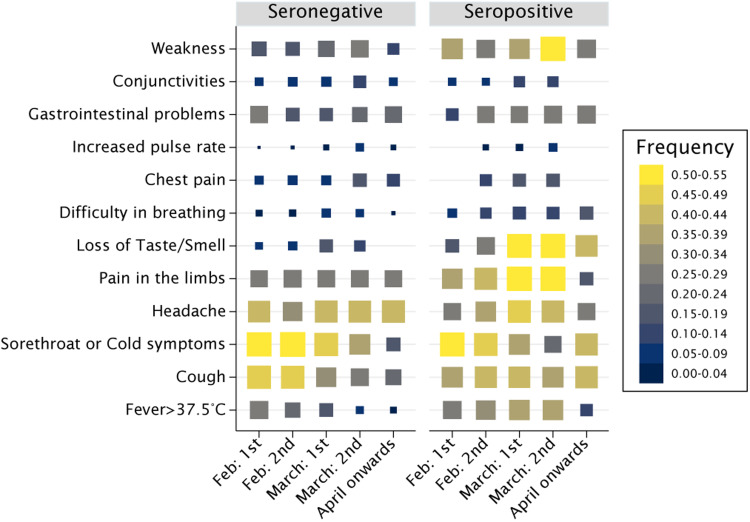
Reported symptoms by time of symptom onset in seronegative and seropositive participants. Rectangle sizes are proportional to the frequency of symptoms across periods, within groups. The symptoms most predictive of seropositivity (loss of taste or smell; weakness; pain in the limbs; fever; and breathlessness) were apparently more prevalent among seropositive than seronegative participants at the time of peak incidence of the epidemic first wave in the valley, between late February, throughout March and part of April.

Among participants with any number of symptoms (*n* = 1002), seroprevalence peaked in those with symptoms onset in the first half of March and returned the second highest figure for onset in the following fortnight (Table 4, all *P* < 0.001). Seroprevalence was higher also among those reporting symptoms in the second half of February, compared to other periods (*P* < 0.001). This curvilinear trend was even more apparent when restricting the analyses to participants reporting 2+ symptoms (*n* = 680), whereas seroprevalence peaked at 63.2% (95% CI 58.1–68.0) among multi-symptomatic participants with reported onset in the first half of March (Table 4, Fig. 3).

Of those participants reporting multiple symptoms (2+), 34.6% (95% CI 31.6–37.8) also reported having sought medical assistance (mostly by contacting the general practitioner) while 65.4% (95% CI 62.2–68.4) had not. Participants with any number of symptoms were more frequently testing positive (51.4%) if they had contacted the healthcare service for their symptoms than if they did not (33.9%, *P* < 0.001), despite the minority (286 *vs.* 716) had sought contact.

We further investigated associations with reported pre-existent chronic conditions (Supplementary Table S4). We observed mild evidence of association with metabolic diseases (OR 0.46; 95% CI 0.22–0.98; *P* = 0.043) and liver disease (OR 2.83; 95% CI 1.00–7.94; *P* = 0.049) in opposite directions. As for regular therapies, directions of association were fairly consistent with those of chronic conditions (Supplementary Table S5). There was mild to weak evidence of negative associations of seropositivity with diabetes therapies (OR 0.38; 95% CI 0.13–0.92; *P* = 0.032) and use of sedatives, antidepressants or antipsychotics (OR 0.57, 95% CI 0.32–1.03; *P* = 0.064). Having had any prior health event was not associated with seropositivity (Supplementary Table S6). However, some reported events such as organ transplantation, chemo/radiotherapy and disautonomy were relatively infrequent to allow drawing definitive conclusions regarding these entities. Among incident health-related issues, or pre-existing issues worsening their clinical course after March 2020, musculoskeletal disorders (OR 2.00; 95% CI 1.38–2.92), respiratory or pulmonary diseases (OR 2.44; 95% CI 1.42–4.20) and sleep disorders (OR 1.81; 95% CI 1.21–2.70) were more frequent in seropositive than seronegative participants (Supplementary Table S6).

## Discussion

The present study shows that nearly 30% of the Gardena valley general population was infected by SARS-CoV-2 during the first pandemic wave. Neutralising antibody capacity was measurably higher than estimated seropositivity. Our in-depth analysis of SARS-CoV-2 determinants of infection as well as COVID-19-related symptoms identified specific seroprevalence risk factors and extensively characterised the post-infection symptoms.

The main strength of the study is its representativeness that guarantees generalisability of the findings to the whole population of the valley. The study was concluded in a short time span of 2 weeks of a nearly 3-month-long strict national lockdown. The whole framework of recruitment, sample-handling and storage procedures allowed the remarkable participation response, while preventing the possible influence of additional external factors through the period.

The present study also comes with several limitations. First, antibody testing methods have imperfect accuracy. We corrected seroprevalence analyses for the test sensitivity and specificity to overcome this limitation. Second, certain social groups such as, for instance, non-Italian residents might have been underrepresented. While we corrected all analyses for differential participation in known groups, we could not prevent participation bias of unknown sources such as, for example, COVID-19-related mortality and possible self-selection of severely ill individuals. A third limitation is the questionnaire self-administration: while this was the only way to collect essential information, response bias might affect some analyses. For instance, symptom onset estimation might not be totally accurate as the question was not specific to each possible symptom. Similarly, we cannot exclude that symptoms were due to competitive seasonal diseases such as flu or allergies. Another potential limitation of our assessment is that the serum antibody assays evaluated against the neutralisation titres could be sensitive to the ratio of asymptomatic-to-symptomatic participants. However, our selection of 299 samples was independent of the symptomatic status. We also performed two resampling strategies, implicitly varying the relative proportion of IgG-positive and IgG-negative among the 299 serum samples for selection, which gave similar sensitivity and specificity results. Lastly, in exploratory analyses comparing IgG ratios across symptomatic groups by serum positivity, there was no evidence that immunoassay test accuracy would differ across different symptomatic groups, as previously reported [13].

A nearly 30% seroprevalence is a large figure compared to other studies [4]. This estimate aligns with those of nearby Italian regions [14, 15]. Reports of SARS-CoV-2 infections in Manaus (Brazil) showed that antibodies detection may underestimate previous infection mainly because of waning [16]. This and other reasons suggest that the true seroprevalence in the current study is also underestimated. First, the survey had missed people who already died. In 2020, the raw excess all-cause mortality rate over the previous 5 years was between+32.8% and +72.4% in the three municipalities [17]. Second, antibodies can persist until 5 months or more, but we cannot exclude that IgG levels had already waned for individuals with earlier or less severe infection or less efficient immune response [18, 19]. Importantly, even in the absence of detectable IgG levels, COVID-19 patients may develop robust T-cell-mediated immune response, with SARS-CoV-2-specific T cells showing a stem-like memory profile over the full disease severity spectrum [20–22]. SARS-CoV-2-specific T cells are missed by the current investigation, contributing to prevalence underestimation. Third, it is debated whether previous exposure to other coronaviruses causing the common cold could generate long-lasting SARS-CoV-2-targeting antibodies. Cross-reactive antibodies were recently identified in few adults and more frequently in children and adolescents supporting pre-existing immunity [23]. Additionally, multiple studies reported cross-reactive T-cell memory in 28–50% people [24], increasing the possibility that some pre-existing immunity is already present. Fourth, adding to the extant evidence, our comparison of the Abbott SARS-CoV-2 assay against the Diasorin assay and the PRNT reflects that a relatively higher proportion of individuals may have been in contact with the SARS-CoV-2.

The imperfect agreement between the Abbott and Diasorin tests can be explained by the two assays being directed against different viral antigens with different kinetics: the anti-nucleocapsid protein IgG for Abbott and the anti-S1/S2 portions of spike protein IgG for Diasorin [19]. Furthermore, the comparison against a neutralisation test identified several samples with neutralisation capacity that had negative antibody test results. Part of their misclassification against the neutralisation test was due to the initial definition of precautionary thresholds set by companies, where unclassifiable/dubious indices were set to negative. The recent revision of diagnostic antibody test thresholds will limit this misclassification [25]. The neutralisation assay was used as a reference to assess previous exposure to SARS-CoV-2. There is also a possibility that antibodies to the nucleocapsid antigen protein develop in the absence of neutralising antibodies. However, only three out of 299 samples with immunoassay-positive result by either test returned no neutralisation titres in our assessment, potentially due to contamination.

According to the reported symptoms, cases of infection have seemingly peaked during the first half of March 2020, as in neighbouring regions [3, 14, 15]. Spoken language, nationality and educational level were not associated with SARS-CoV-2 seropositivity, supporting the absence of social stratification in the exposure to the virus. Sex was not a major determinant of infections even though females had ~20% lower risk than males, in line with a population study conducted in the nearby province of Trento [15]. This might reflect a higher prevention-prone behaviour of females or a higher rate of neutralising autoantibodies against type I interferon in COVID-19 severely affected males [26]. In contrast to similar studies [14, 15], while seropositivity had no general evidence of positive association with age, it was inversely associated with age in the absence of fever and weakness in the present study. This is perhaps due to a socially diverse population of positive individuals, which were younger and arguably linked to winter-sporting activities in this study. Moreover, the association of seroprevalence with municipality and the accommodation and catering services suggests that infections might have been mainly driven by unavoidable occupational circumstances.

Only a minority of symptomatic individuals sought medical advice. While the experience of non-life-threatening symptoms was plausible, we cannot exclude neglect or reluctance to endure quarantine by some citizens if found positive. This poses the question to the transparency and effectiveness of public authorities' communication efforts as well as to individual behaviours.

The apparent protective effect of current smoking on SARS-CoV-2 seroprevalence is not novel [27, 28]. A collider bias effect may supersede sample representativeness [29]. COVID-19 increases mortality risk just as smoking does. Current smoking is causally related to more severe COVID-19 disease [30]. With COVID-19 and smoking intertwined to affect mortality and participation, it is likely that the apparent protective effect of smoking on seropositivity is explained by harvesting effects on mortality, or impairment to participation by health conditions or health-prone behaviours [31].

In conclusion, we confirm that the Gardena valley had one of the highest prevalence of SARS-CoV-2 infection in Europe. Comparisons between distinct antibody detection assays and between serum assays and serum antibodies neutralising capacity, yet suggest an underestimation of actual seroprevalence in this report. While age and sex appear consistently related to infection in other contexts, in settings of high incidence rates possibly linked to the ongoing touristic season in the present case, these demographic factors may exert less prominence than the social context. In contrast, all investigated flu-like symptoms were predictive of a positive antibody test result, with the highest and cumulative evidence for the loss of taste or smell, fever, difficulty in breathing, pain in the limbs and weakness. However, some symptoms were associated with the seroprevalence in an age-dependent mode.

Overall, findings highlight that the determinants of SARS-CoV-2 infection and outcomes are context-dependent, as they relate to the pattern of infection, the local population composition and the economic dynamics. Thus prevention strategies may be tailored to the social context.

## Data Availability

The data that support the findings of this study are available on request from the corresponding authors. The data are not publicly available due to privacy and ethical restrictions.

## Supplementary material

For supplementary material accompanying this paper visit http://dx.doi.org/10.1017/S0950268821001886. click here to view supplementary material

## References

[1] Gudbjartsson DF (2020) Spread of SARS-CoV-2 in the Icelandic population. New England Journal of Medicine 382, 2302–2315. 10.1056/NEJMoa2006100 PMC7175425 32289214

[2] Lipsitch M, Swerdlow DL and Finelli L (2020) Defining the epidemiology of Covid-19 – studies needed. New England Journal of Medicine 382, 1194–1196. 10.1056/NEJMp2002125 32074416

[3] Lavezzo E (2020) Suppression of a SARS-CoV-2 outbreak in the Italian municipality of Vo’. Nature 584, 425–429. 32604404 10.1038/s41586-020-2488-1 PMC7618354

[4] Rostami A (2021) SARS-CoV-2 seroprevalence worldwide: a systematic review and meta-analysis. Clinical Microbiology and Infection 27, 331–340. 33228974 10.1016/j.cmi.2020.10.020 PMC7584920

[5] Bryan A (2020) Performance characteristics of the Abbott architect SARS-CoV-2 IgG assay and seroprevalence in Boise, Idaho. Journal of Clinical Microbiology 58, e00941-20. 32381641 10.1128/JCM.00941-20 PMC7383515

[6] Rodon J (2019) Blocking transmission of Middle East respiratory syndrome coronavirus (MERS-CoV) in llamas by vaccination with a recombinant spike protein. Emerging Microbes & Infections 8, 1593–1603. 31711379 10.1080/22221751.2019.1685912 PMC6853226

[7] Bonelli F (2020) Clinical and analytical performance of an automated serological test that identifies S1/S2-neutralizing IgG in COVID-19 patients semiquantitatively. Journal of Clinical Microbiology 58, e01224-20. 32580948 10.1128/JCM.01224-20 PMC7448652

[8] Lin LI (1989) A concordance correlation coefficient to evaluate reproducibility. Biometrics 45, 255–268. 2720055

[9] Martin Bland J and Altman D (1986) Statistical methods for assessing agreement between two methods of clinical measurement. Lancet 327, 307–310. 2868172

[10] Fleiss JL, Levin B and Paik MC (2003) Statistical Methods for Rates and Proportions, 3rd Edn. New Jersey: John Wiley & Sons.

[11] Youden WJ (1950) Index for rating diagnostic tests. Cancer 3, 32–35. 15405679 10.1002/1097-0142(1950)3:1<32::aid-cncr2820030106>3.0.co;2-3

[12] Rogan WJ and Gladen B (1978) Estimating prevalence from the results of a screening test. American Journal of Epidemiology 107, 71–76. 623091 10.1093/oxfordjournals.aje.a112510

[13] Public Health England (2020) Evaluation of sensitivity and specificity of four commercially available SARS-CoV-2 antibody immunoassays.

[14] Pagani G (2020) Seroprevalence of SARS-CoV-2 significantly varies with age: preliminary results from a mass population screening. Journal of Infection 81, e10–e12. 10.1016/j.jinf.2020.09.021 PMC7836634 32961253

[15] Stefanelli P (2021) Prevalence of SARS-CoV-2 IgG antibodies in an area of northeastern Italy with a high incidence of COVID-19 cases: a population-based study. Clinical Microbiology and Infection 27, 633 e631–633 e637. 10.1016/j.cmi.2020.11.013 PMC7695553 33253941

[16] Buss LF (2021) Three-quarters attack rate of SARS-CoV-2 in the Brazilian Amazon during a largely unmitigated epidemic. Science 371, 288–292. 33293339 10.1126/science.abe9728 PMC7857406

[17] Grafici interattivi sui decessi. Available at https://www.istat.it/it/archivio/241428 (Accessed 02/24/2021).

[18] Chen Y (2020) Quick COVID-19 healers sustain anti-SARS-CoV-2 antibody production. Cell 183, 1496–1507. e1416. 33171099 10.1016/j.cell.2020.10.051 PMC7608032

[19] Dan JM (2021) Immunological memory to SARS-CoV-2 assessed for up to 8 months after infection. Science 371, eabf4063. 33408181 10.1126/science.abf4063 PMC7919858

[20] Neidleman J (2020) SARS-CoV-2-specific T cells exhibit phenotypic features of helper function, lack of terminal differentiation, and high proliferation potential. Cell Reports Medicine 1, 100081. 32839763 10.1016/j.xcrm.2020.100081 PMC7437502

[21] Rodda LB (2021) Functional SARS-CoV-2-specific immune memory persists after mild COVID-19. Cell 184, 169–183. e117. 33296701 10.1016/j.cell.2020.11.029 PMC7682481

[22] Sekine T (2020) Robust T cell immunity in convalescent individuals with asymptomatic or mild COVID-19. Cell 183, 158–168. e114. 32979941 10.1016/j.cell.2020.08.017 PMC7427556

[23] Ng KW (2020) Preexisting and de novo humoral immunity to SARS-CoV-2 in humans. Science 370, 1339–1343. 33159009 10.1126/science.abe1107 PMC7857411

[24] Sette A and Crotty S (2021) Adaptive immunity to SARS-CoV-2 and COVID-19. Cell 184, 861–880. 33497610 10.1016/j.cell.2021.01.007 PMC7803150

[25] Seow J (2020) Longitudinal observation and decline of neutralizing antibody responses in the three months following SARS-CoV-2 infection in humans. Nature Microbiology 5, 1598–1607. 10.1038/s41564-020-00813-8 PMC7610833 33106674

[26] Bastard P (2020) Autoantibodies against type I IFNs in patients with life-threatening COVID-19. Science 370, eabd4585. 32972996 10.1126/science.abd4585 PMC7857397

[27] Carrat F (2020) Seroprevalence of SARS-CoV-2 among adults in three regions of France following the lockdown and associated risk factors: a multicohort study. *MedRxiv* 2020.2009.2016.20195693.

[28] Ward H (2020) Antibody prevalence for SARS-CoV-2 following the peak of the pandemic in England: REACT2 study in 100,000 adults. *MedRxiv* 2020.2008.2012.20173690.

[29] Griffith GJ (2020) Collider bias undermines our understanding of COVID-19 disease risk and severity. Nature Communications 11, 5749. 10.1038/s41467-020-19478-2 PMC7665028 33184277

[30] Ponsford MJ (2020) Cardiometabolic traits, sepsis, and severe COVID-19: a Mendelian randomization investigation. Circulation 142, 1791–1793. 32966752 10.1161/CIRCULATIONAHA.120.050753 PMC7594537

[31] Verlato G (2010) Asthmatics and ex-smokers respond early, heavy smokers respond late to mailed surveys in Italy. Respiratory Medicine 104, 172–179. 19857946 10.1016/j.rmed.2009.09.022

